# Animal welfare organisations that rehome dogs from southern and eastern Europe to Germany: A homepage content analysis

**DOI:** 10.1017/awf.2025.10044

**Published:** 2025-10-13

**Authors:** Jessica Graf, Franziska Kuhne

**Affiliations:** Department Applied Ethology and Animal Behaviour Therapy, Faculty of Veterinary Medicine, Justus-Liebig-University, Frankfurter Strasse 110, 35392 Giessen, Germany

**Keywords:** Animal welfare, cross-border adoption, dog transport, international rehoming, rescue dogs, stray dogs

## Abstract

Each year, over 100,000 dogs are imported into Germany from other EU countries by animal welfare organisations, mainly from Romania. This study conducted a systematic content analysis of websites belonging to 241 animal welfare organisations that rehome dogs from southern and eastern Europe to Germany. Assessment included transparency, legal compliance, and availability of educational and medical information for adopters. The study shows that many organisations lacked information regarding dogs’ origins, transport methods, or health status which sometimes makes it difficult to distinguish between dog rescue and illegal dog trade. Photos were mainly used in adoption advertisements and behavioural descriptions were only included in two-thirds of the dog advertisements which poses a risk of increased dog relinquishment post-adoption due to behavioural unsuitability. Information on vector-borne diseases and typical behaviour of imported rescue dogs was not provided comprehensively. Few organisations violated legal standards, offering underage or banned breeds and failing to use the TRACES transport system. Most organisations relied upon private foster homes, while few had no temporary housing available in Germany. Although most claimed to conduct pre-adoption checks, comprehensive contract details were rarely published. The number of existing animal welfare organisations that rehome dogs from southern and eastern Europe to Germany is unable to be determined due to high fluctuation and the lack of central registry. Inadequate health disclosures and behavioural descriptions risk poor adoption matches and increased returns. Lack of legal compliance may endanger both animal and public welfare and opens the door to illegal dog trade. Sustainable animal protection requires better adopter education, reliable medical testing, and local engagement in source countries to reduce reliance upon transnational rehoming.

## Introduction

Each year, more than 100,000 dogs are brought to Germany by animal welfare organisations from southern and eastern Europe (Graf & Kuhne 2023). Many countries of the eastern and southern European Union face large populations of free-roaming dogs. Due to different social and economic backgrounds, the implementation of animal welfare standards, population control and well-being of dogs shows disparities between those regions and western parts of the EU (Dalla Villa *et al.* 2010). In some countries, including Romania, it is not easy to implement neuter and release programmes for population management, and rehoming of dogs is difficult. Living condition in public shelters are often poor (Pencea & Brădățan 2015) and, consequently, many animal welfare organisations rehome dogs from these countries to other parts of Europe in order to improve their welfare.

The imported dogs are predominantly of mixed-breed who are captured on the street or taken in from poor sheltering conditions by animal welfare organisations and prepared for transport to Germany in foreign animal shelter-like facilities. They are brought to Germany by plane or road transport and either handed over directly to new owners or initially placed in foster homes, from where they are then rehomed.

Previous studies have shown that many of these dogs carry infections and pose the threat of introducing vector-borne diseases to central and western European countries (Schäfer *et al.* 2019). Also, violation of legal health and vaccination requirements increase the risk of introducing rabies to Germany (Klevar *et al.* 2015). The dogs have different social backgrounds that may have led to socialisation deficiencies that can result in behaviour problems post-adoption (Fitzi-Rathgen & Niederer 2018; Norman *et al.* 2020; Graf & Kuhne 2025). Severe behavioural disorders can impair a dog’s welfare which counters the intentions of the animal welfare organisations to improve the dogs’ quality of life (Beerda *et al.* 1997; Collins *et al.* 2022). Although, in general, the prevalence of severe behaviour problems appears to be low, dogs from eastern Europe often show significantly more anxiety- and aggression-associated problems and dogs from southern Europe commonly display prey drive and hunting behaviour, which owners need to take into account when adopting an imported dog (Graf *et al.* 2025).

The majority of imported dogs are acquired via the internet and are not met by their future owners prior to adoption (Graf & Kuhne 2025). The information provided on rescue organisations’ websites is therefore of prime importance regarding future dog owners’ decision to adopt or not. The information, images and video material presented can influence the purchasing behaviour of future dog owners and have a greater impact on the adoption decision than suitability of the dog to the adoptant’s lifestyle (Kelling *et al.* 2024). Choosing a dog without first getting to know it and without knowing the dog’s behaviour and character can lead to an increase in the number of dogs being relinquished post-adoption due to problems in everyday life and undesirable behaviour (Nakamura *et al.* 2020). The homepages are therefore a suitable tool for investigating what information is made available to adopters in their decision-making process and for evaluating the transparency of actions and procedures. Although animal welfare organisations often also make use of social media and online sales portals for the rehoming of dogs, information there is scant and little reference is made to the websites. The analysis of the homepages was therefore chosen as an instrument to examine the work of the organisations.

To date, it is unknown how many dog welfare organisations rehome dogs from eastern and southern European countries to Germany. The aim was not only to determine the number of such animal welfare organisations existing in Germany and the number of dogs available for adoption, but also to take a closer look at the way they are presented and the processes involved in adoption, transport and communication with future owners. In addition to quantifying the number of dogs on offer, the aim was to examine the education and information available to interested parties on the websites as this can have a major impact on the dog’s welfare post-adoption. Furthermore, potential violations of current German and EU laws regarding animal welfare, animal health and transport were explored. The transparency of the organisations’ work and information provided for adopters were also a topic of the content analysis of the homepages. For the purposes of this study, only welfare organisations involved with dogs from countries within the European Union were assessed.

## Materials and methods

### Analysis of organisation numbers and website selection

In order to be able to objectively assess not only the number of organisations but also their transparency and activities, the homepages of the organisations were to be analysed via a systematic content analysis (Früh 2017). This is a qualitative method of empirical social research where data (in this case: websites) are analysed according to a deductive categorical system. To define the sample, only those welfare organisations listed in the German register of charity associations (so-called ‘e.V.’) were considered. Other welfare organisations, e.g. those not legally recognised as non-profit, those operating from outside Germany and private individuals that rehome imported dogs to Germany, were not considered in this analysis because it is not possible to objectively identify all of them. The German register of charity associations is publicly accessible (www.handelsregister.de, Ministry of Justice of the State of North Rhine-Westphalia, Düsseldorf, Germany), but it is not possible to carry out a filter by area of activity and it contains information pertaining to millions of different charities and non-profit organisations. As it had to be assumed that many organisations would possess abstract and creative names and, thus, would not have been included in a search request using a definite number of keywords, a systematic analysis of all charity’s names throughout the entire register was carried out in the period from October 2021 to February 2022.

In order to recognise an organisation listed in the German register of charities as an animal welfare organisation, the name had to contain indications that the organisation worked in the area of animal welfare, for example ‘animal welfare organisation’, ‘strays’, ‘paws’, ‘animal aid’, etc. If such an organisation was identified, a search engine query (www.google.de, Google LCC, Mountain View, CA, USA) was used to search for a website. If it was recognisable from the homepage or social media presence (e.g. Facebook, Instagram) that the organisation predominantly (more than 50% of all dogs on offer) offered dogs from southern and eastern Europe for adoption in Germany, it was counted as being a welfare organisation that qualified for analysis. As a result of this procedure, animal welfare organisations not publicly offering their dogs on the internet were not able to be identified and therefore not included in the overall count of the number of dog welfare organisations. Organisations that only presented dogs from other German organisations as so-called ‘adoption assistance’, but were not directly responsible for their adoption, were not included. Municipal or communal animal shelters, which are mainly responsible for the care of lost and abandoned animals, were also not taken into account, except when more than half of the dogs on offer were described as having come from other European countries.

In total, 764 dog welfare organisations were identified via this analysis. Subsequently, a representative sample of 350 organisations was randomly selected from the organisations found, and their homepages subjected to a systematic qualitative content analysis according to Früh (2017). For the selection of organisations for the analysis, each of the 764 organisations was given a number in order of its appearance on the charity register. Using a web tool for random selection (random.org, Randomness and Integrity Services Ltd, Dublin, Ireland), 350 organisation numbers were randomly selected to constitute the sample.

#### Content analysis

According to Früh (2017), a coding manual was developed by defining several theses of interest and creating categories of available homepage information to accept or decline them. For the analysis, all available information on the websites were used, including all text data as well as photos and videos of dogs and downloadable resources (e.g. contracts, guidelines). External links were not followed. In order to test the practicability and reliability of the code book, a pre-test was first carried out with a sample of 16 randomly selected organisations, which was completed by the researcher and seven other people in order to verify the reliability of the analysis. The trial coders were all volunteering individuals working in the veterinary field but not previously involved in social research. They received a training session during which the technique was explained to them by the researcher with whom they carried out joint analysis of an example homepage prior to addressing the pretest without further assistance. For intercoder reliability testing, Holsti’s method was used, with a reliability value greater than 0.75 considered as acceptable for categories, according to Holsti (1969). Otherwise, the category was excluded from the analysis. The coding manual used for the analysis was then adapted to the results of the pretest and is available in the Supplementary material. The actual analysis was then performed by the researcher and carried out between September 2022 and July 2023.

The data collected were subjected to a descriptive statistical analysis. In addition, a single repeated-measures ANOVA was performed to evaluate the development of the number of offered dogs over time. Microsoft Excel 2019 ® (Microsoft Corporation, Redmont, WA, USA) was used for data collection and the statistical analyses were performed with IBM SPSS ® Version 28 (SPSS Inc, Chicago, IL, USA).

### Ethical statement

Ethical approval was not required to carry out this study because the systematic review only collected data from publicly available sources. Data on animal welfare organisations were collected through a publicly available source (www.handelsregister.de, Ministry of Justice of the State of North Rhine-Westphalia, Düsseldorf, Germany). For the random sample of organisations that were accepted into this study, no names or identification numbers were collected to ensure anonymity.

## Results

### Analysis of the register of charity associations

**Figure 1.**
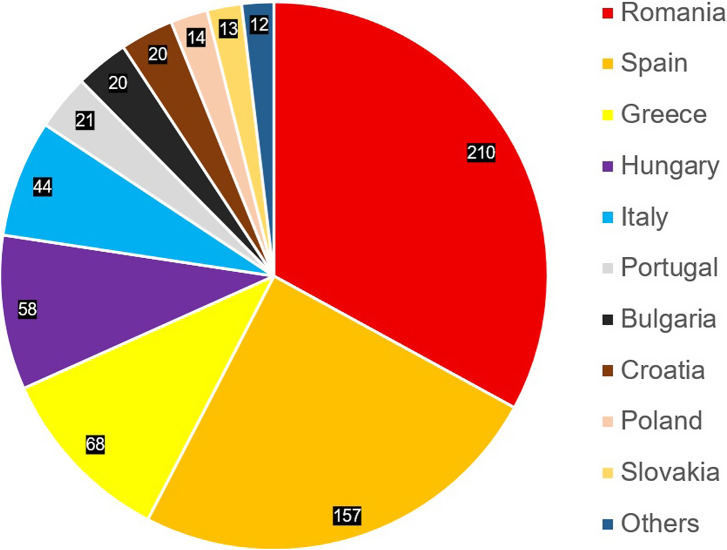
Numbers of dog welfare organisations that rehome dogs from respective countries to Germany (n = 637). For 130 organisations it could not be clearly identified from which EU country the dogs were imported from, and these are not included in the graph.

By analysing the German Register of Associations, a total of 764 registered organisations based in Germany were found that offered dogs from southern and eastern Europe for adoption in Germany and were recognisable as such organisations via their websites. Most of the organisations were based in North Rhine-Westphalia (n = 187; 24.5%) and Bavaria (n = 123; 16.1%). It was not possible to determine from which European country dogs were being offered for adoption from for all the organisations. A total of 135 of the organisations (17.7%) offered dogs from different countries, with occasionally dogs from non-EU countries such as Ukraine, Bosnia or Serbia also being offered. Around one-third offered dogs from Romania. Figure 1 shows the number of organisations by country of origin of the dogs offered for adoption.

### Analysis of the organisation homepages

Data from 241 of the 350 randomly selected organisations were available for analysis (68.9%). The remaining organisations (n = 109; 31.1%) either no longer existed at the time of the analysis (n = 11; 3.1%), did not offer dogs from other EU countries at the time of the analysis (n = 20; 5.7%), did not place dogs themselves (n = 15; 4.3%) or most frequently had no current or no website at all outside of social media (n = 63; 18.0%) that could be used for the analysis. Organisations based in North Rhine-Westphalia (n = 65; 27.0%) and Bavaria (n = 33; 13.7%) were also most frequently represented in the analysis. No organisations from Bremen or Thuringia were included in the selection (Table 1).

**Table 1.** Number of dog welfare organisations found in total in the German registry of charities and number of dog welfare organisations in the analysed sample, respectively by federal state in which the organisation is based

Federal State	Organisations in total	Organisations in analysed sample
*North Rhine-Westphalia*	187	65
*Bavaria*	123	33
*Baden-Wuerttemberg*	105	41
*Hesse*	81	26
*Lower Saxony*	77	23
*Rhineland-Palatinate*	69	21
*Schleswig-Holstein*	29	4
*Brandenburg*	21	5
*Berlin*	17	7
*Saarland*	16	4
*Hamburg*	11	5
*Mecklenburg-Western Pomerania*	9	3
*Saxony*	8	2
*Thuringia*	6	0
*Saxony-Anhalt*	3	2
*Bremen*	2	0
***Total***	**764**	**241**

The median foundation year of the organisations was 2012, with the oldest having been in existence since 1841 and the youngest founded in 2022.

**Figure 2.**
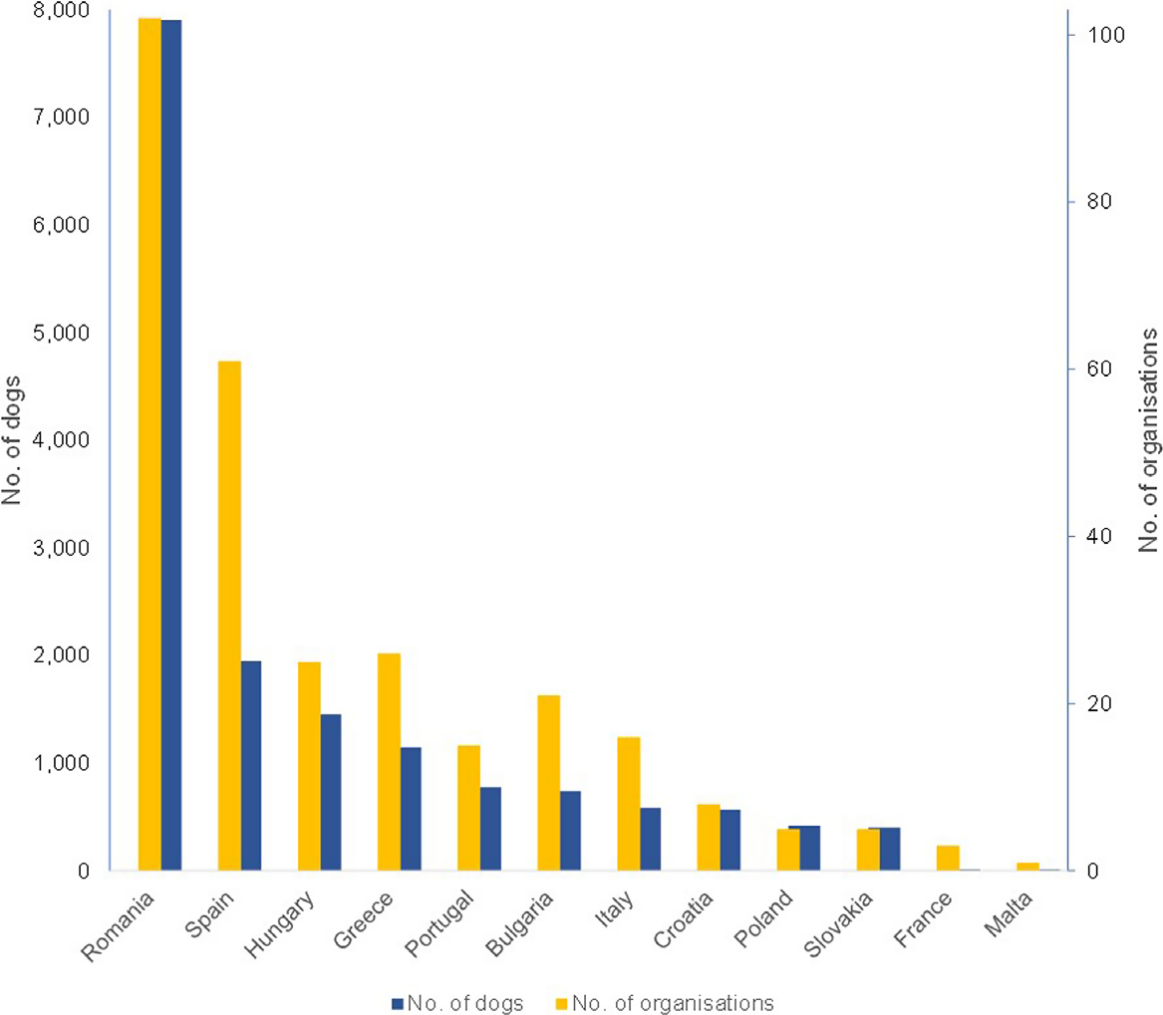
Number of dogs offered for adoption (blue) and number of dog welfare organisations (orange) by country of origin.

Of the organisations in the sample, 41 out of 203 (20.2%) rehomed dogs from several countries. Most of the dogs came from Romania and most of the organisations also rehomed dogs from Romania (Figure 2).

The number of dogs rehomed increased significantly in the survey period from 2018 to 2021 (*F*_2, 103_; 124.068 = 5.366; *P* = 0.005). Table 2 shows the mean number of dogs rehomed for each year and the maximum number of dogs rehomed by a single organisation in each year.

**Table 2.** Mean, minimum, and maximum number of dogs rehomed per welfare organisation in the period 2018–2021

Dogs rehomed (year)	Organisations with information available (N)	Mean	Minimum	Maximum
2018	70	90.1	1	324
2019	76	111.2	1	927
2020	83	143.0	2	1,528
2021	87	153.3	1	2,400

At the time of the analysis, an average of 77.5 dogs per organisation were currently available for adoption, with the number varying from zero dogs to 1,294 dogs per organisation.

The sex ratio of the dogs offered for adoption was balanced (48.8% female and 51.2% male). Most of the dogs offered were aged between one and eight years (62.5%), but almost one-quarter were younger than one year (23.7%). Dogs over eight years of age made up a smaller proportion (13.7%).

The mean (± SD) adoption fee, where stated, averaged €396.39 (± 56.59).

A character description of the dogs on offer was available from 68.5% of the organisations, 93.8% of the organisations showed photos of the available dogs and 25.3% also had videos of the majority of the dogs on display.

The proportion of dogs with a disease or infection already stated by the organisation or visible in a dog’s pictures in the online offer was in the single-digit percentage range (Table 3).

**Table 3.** Diseases and infections listed on the welfare organisations’ homepages for dogs available for adoption in absolute numbers (mean [range]) and as a mean proportion of total dogs offered per organisation

	Dog per Organisation	% Dogs per Organisation
Diseases of the musculoskeletal system	4.2 [1–52]	5.7%
Neurological diseases	2.2 [1–13]	3.7%
Internal diseases	3.1 [1–21]	4.6%
Amputations	3.9 [1–90]	4.3%
Other diseases	3.1 [1–33]	5.0%
Infectious diseases	6.2 [1–44]	8.5%
- Leishmaniasis	4.2 [1–35]	8.4%
- Other VBDs	5.1 [1–34]	6.9%
- Other infections	3.4 [1–20]	3.2%

VBD = Vector-borne diseases.

Overall, 132 out of 208 (63.5%) of the organisations stated that they had tested their dogs for vector-borne infections prior to leaving for Germany. The type of test and which pathogens were included were not recorded.

The majority of the organisations (64.6% of 175) transported their dogs to Germany by road, with only 17.7% using aircraft. A further 17.7% of the organisations used both road and air transport and only 83 out of 144 (57.6%) of the organisations clearly indicated that the transport was carried out exclusively by commercial transporters. Almost one-third of the organisations (31.3% of 144) also used private individuals as flight patrons. These are private individuals who transport dogs to Germany on behalf of the organisation when they return from trips from other European countries so that the dogs can then be handed over to their new owner or temporarily housed in a foster home.

No information on transportation could be found for 97 organisations.

Two of the surveyed animal welfare organisations offered dogs that were under the age of 15 weeks for immediate adoption, meaning that they could not have been legally transported to Germany. Five of the organisations offered dogs that were unfit for transport due to health reasons. In 14 organisations, Pitbull Terriers, American Staffordshire Terriers, Staffordshire Bullterriers, Bullterriers or crossbreeds of those were offered for adoption. Section 2 (1) of the Act on the Restriction of the Introduction or Import of Dangerous Dogs into Germany (HundVerbrEinfG) stipulates that it is illegal to bring these breeds of dog into Germany. The homepages of 44 organisations did not indicate that the dogs were transported via the TRACES (European Trade Control and Expert System) system.

The organisations mainly used a network of private foster homes for temporary housing of dogs in Germany (82.1% of 229 organisations); only very few organisations had their own animal shelter in Germany (7.9%) or a partner animal shelter (2.6%) available. Some organisations (7.4%) had no possibility of providing temporary shelter for dogs in Germany.

In 71.8% of the organisations, a pre-adoption check was mandatory before adopting a dog, while 24.9% did not provide any information on this. Only 33.6% of the organisations provided general information about common behavioural traits of imported dogs on their websites, 65.6% did not. General information on vector-borne diseases was also only displayed on 38.6% of the websites and was missing on 60.6%.

Detailed information regarding the regulations in the adoption contract was only publicly available for a few organisations (n = 54). In 25 of these organisations, a contractual obligation to neuter was identified. Two organisations strictly rejected conventional medical care for the dogs, in one case training was advocated that was detrimental to animal welfare and in two organisations rehoming to tethering or kennel-keeping was possible.

## Discussion

### The organisations

The results of the study show that it is difficult and not very transparent to map the landscape of organisations in the field of international dog rehoming. Currently it is impossible to obtain an overview of all the organisations that bring dogs from other European countries to Germany. The register of associations does not offer the option of filtering a search of organisations according to their activities. It must therefore be assumed that the actual number of organisations is substantially higher than the 764 identified in this study. Animal welfare organisations sometimes give themselves creative names that do not necessarily allow their activities to be deduced, which means that they may have been overlooked when searching the database. Welfare organisations that do not offer their dogs publicly via the internet could also not be detected in this analysis. It must also be assumed that numerous private individuals, organisations not recognised as non-profit organisations and other groups of people also bring dogs to Germany from abroad. There are also organisations that are based in the dogs’ country of origin and carry out their rehoming activities from there without being registered in Germany.

Organisations that bring dogs to Germany from abroad require a permit from the competent veterinary office in accordance with Section 11 (1) No 5 of the German Animal Welfare Act (TierSchG). An exact determination of the number of organisations could therefore only be made through the central consolidation of all data from the German veterinary offices which is currently not possible. Each of the 388 veterinary offices in Germany monitors an average of 2.8 (± 3.1) organisations (Graf & Kuhne 2023). Assuming a total number of 388 veterinary offices, this would result in an approximate total of 1,086 organisations in Germany, which is already more than could be determined in this analysis. However, this can only be a rough estimate, and the exact number remains difficult to determine.

Furthermore, the organisations themselves are in a constant state of flux. Around one-tenth were no longer in existence at the time of the analysis – approximately one year after the register of associations was queried – and a further fifth were not currently rehoming dogs at that time.

The organisations were mainly founded in the last twenty years, which indicates that the rescue of animals abroad remains a relatively recent phenomenon. This may be partly due to the liberalisation of the EU internal market and the associated simplification of dog transport within the EU through the introduction of an EU pet passport in 2004 and cross-border regulations. Furthermore, animal welfare has grown in importance societally in recent years with public interest also becoming increasingly focused on practices and animal husbandry abroad that are – from our perspective – contrary to animal welfare. So far, there are no unified legal standards for the welfare and keeping of dogs in the European Union. Romanian law reforms, legalising euthanasia after a short period of sheltering in 2013, have triggered ethical concerns and led many western European organisations to engage in dog welfare and rescue in Romania. Public shelters in Romania are often overcrowded and the welfare of the dogs can be severely compromised with high rates of disease and insufficient space and feeding resources. Stray dog populations are not well tolerated and in urban areas especially animal cruelty often does not lead to prosecution (Pencea & Brădățan 2015; Deutscher Tierschutzbund e.V. 2025).

Overall, this analysis also shows an increase in the number of dogs rehomed in recent years, both before and during the COVID-19 pandemic, in line with the veterinary office staff survey as well as the corresponding TRACES figures (Graf & Kuhne 2023). This may be due to a general increase in the number of pet owners in Germany (ZZF e.V./IVH e.V. 2024). The COVID-19 pandemic saw an especially pronounced increase in the number of dogs in Germany, which meant that more dogs arrived from abroad to meet this demand (TASSO 2020). In addition, dog breeding in Germany has been on a downward trend over the last twenty years, which means that consumer demand for dogs cannot be adequately met (VDH 2024).

The analysis shows that most of the organisations rehome dogs from Romania. This is consistent with the results of the survey of veterinary office employees, the TRACES figures and other studies (Norman *et al.* 2020; European Comission 2023; Graf & Kuhne 2023). It is particularly noteworthy that many organisations offer and rehome dogs from several countries and in some cases, it is not even clear where the dogs come from.

In a large-scale EU enforcement action, it was discovered that some dog traders smuggle dogs from eastern non-EU countries to Romania in order to sell them on to western Europe under the guise of animal welfare (European Commission 2023). The non-transparent online sale of rescue dogs from abroad therefore poses a great risk of illegal dog trade and support of puppy mills where dogs grow up under unacceptable health and living conditions and suffer from deprivation. Organisations that do not offer transparent disclosure of the origin of their dogs and seemingly arbitrarily place dogs from several countries run the risk of their animal welfare activities being called into question and financial profit motives being assumed (Wilczek 2008). It is also apparent that the size of animal welfare organisations varies greatly. While, on average, organisations were offering a few hundred dogs for adoption, some had up to 2,400 dogs on offer. Others had adoption figures in the single-digit range. This also shows how inconsistently the organisations operate and the heterogeneous approach of the animal welfare organisations. As a result, future dog owners are often unable to differentiate between dog rescue and trade and those animal organisations not operating transparently run the risk of having their non-profit welfare activities called into question.

In Germany, animal welfare organisations tend to work in conjunction with a system of private foster homes. In 7.4% of cases, there was no possibility of temporarily accommodating dogs in Germany. However, this can become a necessity at any given time, especially in instances where dogs are relinquished by their new owners. If an organisation is unable to care for such dogs appropriately, they must be placed in a German animal shelter, which adds further burden on animal shelters financed by local authorities and donations (Sippl & Pogner 2025).

One-third of the organisations stated on their homepages that they work with flight patrons. This practice is considered legally controversial since it tends to entail an illegal circumnavigation of the TRACES system (Hillermann & Schatz 2019). In addition, transport in the cargo hold of an aircraft is highly stressful for the dogs and can lead to severe anxiety and trauma (Bergeron *et al.* 2002; Jahn *et al.* 2023). Transport in an aircraft cargo area should therefore be discouraged from an animal welfare perspective.

Scanning websites for specific legal violations, such as the transport of dogs that are too young, the import of dangerous dogs in accordance with Section 2 (1) HundVerbrEinfG and the lack of transportability of the dogs, only produced a few potential breaches.

However, these results are severely limited by a bias, as it must be assumed that violations of the law are hardly ever knowingly communicated publicly and be able to be found on websites.

However, it is striking that 44 organisations made no reference to TRACES documentation when transporting dogs, and this was also a frequent point of criticism in the survey of veterinary office employees (Graf & Kuhne 2023). It is therefore possible that some organisations are still unaware that the transport of dogs by non-profit welfare organisations also constitutes commercial transport and that TRACES documentation is required, as has been clearly stipulated (Europäischer Gerichtshof [Vierte Kammer] 2015).

Ultimately, it was found that the protection or adoption contracts of certain organisations included a ‘compulsory castration’, i.e. the future owners were required to surgically neuter the adopted dog once it reached a certain age in order to prevent it from breeding.

This constitutes an offence under Section 6 (1) TierSchG, as the prevention of reproduction is not a general medical indication for surgical neutering and less-invasive methods are also available.

Although the aim of this study was also to ascertain the extent to which German animal welfare organisations in the dogs’ countries of origin were committed to sustainable animal welfare at a local level, a lack of information on the websites in question made this impossible.

The websites generally contained little or no information regarding activities abroad that sustainably impact animal welfare in the dogs’ country of origin, such as neutering campaigns, education or local rehoming programmes. This suggests that many organisations that rehome dogs in Germany from other European countries have little or no involvement in other animal welfare projects outside of the international rehoming of dogs. Exporting dogs to another country is not a suitable means of sustainably improving the situation of dogs in other parts of Europe. This requires neutering programmes, political commitment and educational work throughout the population (Smith *et al.* 2020).

### The rehoming of dogs

Adult dogs make up the largest group of dogs offered for adoption, but many young dogs and older dogs are also offered. However, a recent survey of dog owners has shown that it is mainly young dogs that are brought to Germany (Graf & Kuhne 2025). From this, we may deduce that young dogs tend to have shorter adoption times and must have a higher turnover rate. The likelihood is that younger dogs are probably advertised for a shorter period of time whilst adult and older dogs’ advertisements are online for a longer period of time, creating a bigger choice of adult dogs and limited availability of younger dogs. Further studies on this topic would be helpful.

The main tool of the adoption adverts on the homepages is pictures of the dogs, which were almost always available. Only roughly two-thirds of examined homepages offered up descriptions of the dogs to be rehomed. It is known from other studies that pet owners often choose their pet based on appearance and that photos are decisive for an adoption choice (Weiss *et al.* 2012; Nakamura *et al.* 2020). However, it is problematic that dogs selected on the basis of their appearance do not necessarily have to be suitable in terms of character. This increases the risk of problem behaviour. According to various studies, undesirable behaviour is the main reason for the return of shelter dogs (Hawes *et al.* 2020; Powell *et al.* 2022). It is also known that the choice of words in a descriptive text can significantly influence the purchase decision (Nakamura *et al.* 2019). Further studies are therefore needed to examine the advertising texts in terms of language choice, validity and truthfulness.

On a positive note, it should be emphasised that pre-adoption checks are regularly carried out by the organisations. Although this information could only be found on around three-quarters of the homepages, it was always stated that a pre-adoption check is carried out on the future owners. This is useful to ensure compliance with animal welfare regulations post-adoption and increases the likelihood of long-term success of the adoption, as the living conditions of the interested parties can be better assessed and this offers an additional opportunity for educational work in the early stages of adopting a dog (Griffin *et al.* 2020). However, the study did not evaluate the rigour and quality of the checks and further studies would be desirable.

The homepages often lack information on vector-borne diseases, which are not yet endemic in Germany and pose a health risk for imported and native dogs. In just over one-third of cases, potential adoptants were able to find information on that on the organisation’s website. The same applies to dealing with the typical behaviour of imported dogs. Eastern European dogs are often mixed breeds from livestock guarding breeds while southern European dogs are often hunting dogs (Graf *et al.* 2025). As a result, depending on the country of origin and the local population, breed-specific behaviours may be displayed which prospective dog owners should be made aware of, to help assist with their preparation. In addition, growing up in shelter-like facilities, little or even negative experience with people and transport over long distances can influence the behaviour of the dogs and may result in an increased requirement for professional dog training (Harvey *et al.* 2016; Dietz *et al.* 2018; Salonen *et al.* 2023; Graf *et al.* 2025).

Diseases and infections were rarely reported in the dogs offered for adoption and were in the single-digit percentage range for both vector-borne infections and other diseases. It must be assumed that there is a very strong bias here and that organisations either do not publicly disclose known diseases *per se* or that tests had not yet been carried out at the time the advertisement was placed. In an owner questionnaire, 37.2% of owners stated that their imported rescue dog was already known to have a disease or infection prior to adoption (Graf & Kuhne 2025).

It is therefore possible that known illnesses and the results of medical tests are only passed on to interested parties in direct contact or that tests are only carried out once adoption of a dog is actually taking place.

In 63.5% of the homepages, it was possible to verify that the dogs had generally been tested for vector-borne diseases prior to entering Germany. This information is consistent with the results of the owner survey, according to which 61.5% of dogs were tested before entering Germany (Graf & Kuhne 2025). It was not possible to determine which type of test and which pathogens were tested for, as the sample size was too small and the homepage information too imprecise. This shows that comprehensive testing of imported dogs before entry into Germany does not take place, although this is recommended (ESCCAP Deutschland e.V. 2018). Young dogs, in particular, are likely to have not been tested, as some animal welfare organisations consider the risk of infection to be low for these dogs, for whom some antibody-testing is not always conclusive. Nevertheless, an infection, for example, with *Leishmania infantum*, can also occur transplacentally or in early puppyhood, which makes testing prior to departure necessary (Naucke & Lorentz 2012).

### Animal welfare implications

The welfare of rescue dogs imported from southern and eastern Europe for adoption in Germany should be of greatest importance to animal welfare organisations that are involved in the rehoming process. The aim of international rehoming rescue dogs is to improve their welfare and protect them from health threats and cruelty in their home countries. Therefore, animal welfare organisations need to show full compliance with existing laws and standards concerning animal welfare to an international standard as well as at national German level. This was not always the case in the studied organisations. It was the aim of this study to evaluate how those organisations provide information for prospective owners throughout the adoption process. The overall welfare of these dogs can be greatly improved by ensuring better management of the importation process, including thorough health checks and behavioural assessments prior to and during transportation. Additionally, providing more detailed information to prospective adopters, especially regarding vector-borne diseases and common behaviour of imported dogs would help them prepare adequately for the needs of their new pets, reducing the likelihood of misunderstandings and increasing the chances of successful long-term adoptions. The lack of transparency in terms of dogs’ origins and rehoming via social media pose the threat of illegal dog trade taking place under the guise of dog rescue. Results of this study and data from the 2023 EU enforcement action on illegal dog and cat trade raise strong concerns for the existence of a lucrative business with commercial dog streams across Europe. For as long as the origin of dogs are not clearly traceable, it appears that the rehoming of dogs from eastern and southern Europe opens the door to illegal trade. Rehoming via online platforms makes dog selling easy and anonymous and customers are blindfolded by tragic storytelling and their wish to help an individual dog. Therefore, animal rescuers should ensure public traceability of the transported dogs as well as transparency regarding their charity work abroad to ensure not only legal action, but also a guarantee of dog rescue that is ethically correct and welfare enhancing.

## Conclusion

In summary, it can be said that the landscape of German animal welfare organisations that rehome dogs from other EU countries is not only in a constant state of flux but also lacks transparency, both of which make it difficult to clearly distinguish animal welfare work from the illegal dog trade. Precise quantification remains impossible due to the lack of a central registration and control body. Our analysis has highlighted that numerous small organisations exist that concentrate on the rehoming of a small number of dogs. However, there are also large to very large organisations that offer numerous dogs and extend their rehoming activities to several countries. The number of unreported cases of those who do not operate as registered organisations but as private individuals or through abroad organisations cannot be estimated and is probably very high.

The dogs on offer were relatively young in age and advertised mainly using pictures. There was not enough information available regarding the common behavioural traits of imported dogs and vector-borne infections. In some cases, violations of applicable animal welfare, animal transport and animal health laws were found on the publicly accessible homepages. This indicates that certain animal welfare organisations are unaware of the correct procedures regarding international dog rehoming.

An analysis of the websites of animal welfare organisations bringing dogs to Germany from southern and eastern Europe shows that many contain very little information and numerous organisations have no assessable website. For this reason, further studies are required to enable an even more in-depth evaluation of the adoption practices of animal welfare organisations. It must be assumed that the majority of adoptions take place almost exclusively via social media. Further research is needed to determine the extent to which a correct description of the dogs, good information for owners and sustainable rehoming practices can be achieved via this channel.

A central registration of animal welfare organisations and a consolidation of information from different cities and districts would be desirable, also in terms of facilitating official monitoring. There is also a need for future legal regulation of the rehoming of dogs via the internet, which could improve the traceability of dogs and transparency for costumers.

## Supporting information

Graf and Kuhne supplementary material Graf and Kuhne supplementary material

## Supplementary material

The supplementary material for this article can be found at http://doi.org/10.1017/awf.2025.10044.
