# Supplementary material for: Animal welfare organisations that rehome dogs from southern and eastern Europe to Germany: A homepage content analysis
Source: Anim Welf. 2025 Oct 13;34:e67. doi: 10.1017/awf.2025.10044 (PMC12554802; doi:10.1017/awf.2025.10044)
Supplement: Graf and Kuhne supplementary material [file S0962728625100444sup001.pdf]

Animal welfare organisations that rehome dogs from southern and eastern Europe to Germany: A homepage content analysis: Supplementary material

Jessica Grafhttps://orcid.org/0009-0009-0978-639X and Franziska Kuhne

Department Applied Ethology and Animal Behaviour Therapy, Faculty of Veterinary Medicine, Justus-Liebig-University, Frankfurter Strasse 110, 35392 Giessen, Germany

Author for correspondence: Jessica Graf, email: jessica.graf@vetmed.uni-giessen.de

Coding manual - Web analysis homepages of animal welfare organisations

Research hypothesis: How welfare-friendly is the transport of rescue dogs from other EU countries to Germany by registered animal welfare organisations?

General instructions for completion

The aim of this study is an objective evaluation of the homepages of animal welfare organisations that bring dogs from other European countries to Germany. For the purpose of objectivity, it is imperative that each coder (that's you!) adheres to the instructions here and does not make decisions 'at their own discretion'. If anything is unclear, you have the opportunity to ask the researcher (that's me!) in the first week (first week of May), the ambiguities will then be added here again and you will all receive an updated version afterwards. Since the purpose of the exercise is to test the criteria set out here for their general applicability, it is very important that you do NOT exchange information with each other! Everyone fills in the form for themselves. Should any questionable aspects arise after the 'trial week', you will be forced to make the most logical decision at your own discretion.

The survey is divided into 4 sections, all of which must be completed for each individual homepage. It is important that you take a complete look at the homepages and, if necessary, download and include any available documents (e.g. protection contracts, information material on diseases, etc.).

If the organisation also places other animal species, only dogs will be considered. Only dogs that are actually placed by the organisation itself will be considered. You will often find dogs from other organisations or similar on the homepages, but these will not be taken into account.

You will receive an Excel table in which one row corresponds to the values of an animal welfare organisation. The columns correspond to the numbers given below. Only numbers are to be entered in the table. You can find out when to enter which number in the tables in these instructions.

You will be given two homepages per week to analyse. If you don't manage both, that's no problem. Just do as much as you can. Please only do the respective homepages in the weeks for which they are entered. No retrospective or premature evaluation!

If nothing can be entered in an Excel field because there is no information on a homepage, please leave this field empty.

1. **General statistical information**

- The organisation
- Number of the organisation

I have included the number of the organisation with the names and links. Please simply copy it here.

- Date of the examination

Please enter the date on which you view the website. If you split it up over several days, simply enter the day on which you start.

- **Location of the organisation [federal state] (nominally scaled)**

Baden-Wuerttemberg	1
Bavaria	2
Berlin	3
Brandenburg	4
Bremen	5
Hamburg	6
Hesse	7
Mecklenburg-Western Pomerania	8
Lower Saxony	9
North Rhine-Westphalia	10
Rhineland-Palatinate	11
Saarland	12
Saxony	13
Saxony-Anhalt	14
Schleswig-Holstein	15
Thuringia	16

You can usually find this information in the imprint!

- Year of foundation of the organisation

How many dogs are brought to Germany by animal welfare organisations?

<u>Hypothesis: The actual number of dogs rehomed significantly exceeds the number of TRACES reports.</u>

1.1.1 Number of dogs placed in 2018 (interval-scaled)

1.1.2 Number of dogs rehomed in 2019 (interval-scaled)

1.1.3 Number of dogs rehomed in 2020 (interval-scaled)

1.1.4 Number of dogs currently available for placement (interval-scaled)

1.1.4.1 Number of female dogs

1.1.4.2. number of male dogs

1.1.4.3. number of dogs under 1 year old

1.1.4.4. number of dogs 1 year to 8 years old

1.1.4.5. number of dogs over 8 years old

When filling in these fields, it may help you to keep tally sheets on a piece of paper while you scan the individual dogs for all characteristics on the homepage. For the sake of simplicity, you can perhaps also combine this with points 2.2. and 3.2.

Example: For a 7-year-old spayed female dog that is already in a foster home in Germany and has leishmaniasis and a severe anxiety disorder, you must then enter a total of 7 ticks on your tally sheet (<8 years [2. 1.4.8.]/neutered [2.1.4.11]/female [2.1.4.1.]/in Germany [2.1.4.9.]/with infectious disease [3.2.1.3.]/with leishmaniasis [3.2.4.2.]/with anxiety disorder [3.2.2.2.2.])!

IMPORTANT: Only fill in what is actually stated and do not make any assumptions! If the neutering status of a dog is not indicated, you cannot enter it anywhere!

ATTENTION - many organisations list the dogs several times in different categories, so you must be careful not to count them twice.

<u>Hypothesis: The country of origin of most dogs is Romania.</u>

<u>Dogs that do not come from any of the listed countries are not taken into account!</u>

1.1.1 Bulgaria [number of dogs] (interval-scaled)

1.1.2 Estonia [number of dogs] (interval scaled)

1.1.3 France [number of dogs] (interval scaled)

1.1.4 Greece [number of dogs] (interval scaled)

1.1.5 Italy [number of dogs] (interval scaled)

1.1.6 Croatia [number of dogs] (interval scaled)

1.1.7 Latvia [number of dogs] (interval scaled)

1.1.8 Lithuania [number of dogs] (interval scaled)

1.1.9. Malta [number of dogs] (interval scaled)

1.1.10. Poland [number of dogs] (interval scaled)

1.1.11. Portugal [number of dogs] (interval scaled)

1.1.12. Romania [number of dogs] (interval scaled)

1.1.13. Slovakia [number of dogs] (interval scaled)

1.1.14. Slovenia [number of dogs] (interval scaled)

1.1.15. Spain [number of dogs] (interval scaled)

1.1.16. Czech Republic [number of dogs] (interval scaled)

1.1.17. Hungary [number of dogs] (interval scaled)

1.1.18. Cyprus [number of dogs] (interval scaled)

Under what conditions is the movement of dogs justifiable?

<u>1.1 Hypothesis: Only dogs that are suitable for a life in Germany are transferred.</u>

To answer the following points, only dogs that are currently available for placement are considered.

1.1.1 Diseases of the dogs

1.1.1.1 Number of dogs with arthrosis/diseases of the musculoskeletal system

1.1.1.2. number of dogs with neurological diseases/legia/paresis

1.1.1.3. number of dogs with infectious diseases

1.1.1.4. number of dogs with internal diseases

1.1.1.5. number of dogs with amputated body parts

1.1.1.6. number of dogs with other diseases (E.g. deafness, blindness)

1.1.2 Number of dogs with infectious diseases

1.1.2.1 Number of dogs with leishmaniasis

1.1.2.2. number of dogs with other VBDs (these include: Ehrlichiosis, Babesia, Anaplasma, Hepatozoon, Dirofilariasis, Lungworm, Rickettsia)

1.1.2.3. number of dogs with other infectious diseases (giardia, parvovirosis, leptospirosis, distemper, HCC)

The diseases listed are all directly or indirectly transmissible to dogs in Germany. This question is therefore NOT only about whether the dog is ill, even if it is a carrier of one of the pathogens mentioned, this must be stated here. This also applies if a disease has supposedly already been overcome (the animals often remain infected for life and may continue to excrete the pathogen).

Dirofilariosis = heartworm or skin worm

Lungworms = Angiostrongylus, Crenosoma

Remember: You can only state what is actually written somewhere!

<u>1.2 Hypothesis: Screening for infectious agents is always carried out before the dogs are moved.</u>

Yes 1

No 0

Screening for leishmaniasis, ehrlichiosis, babesiosis and hepatozoonosis is particularly important here, as these pathogens are not yet endemic in Germany, or not endemic throughout the country. Depending on the country, not all of these pathogens are often tested; the issue here is whether the organisations at least make an attempt to clarify the issue of diseases before bringing them to Germany.

For example, young dogs are often not tested, but adults are, which would then be included.

How animal-friendly is the implementation of the transport by the animal welfare organisations?

1.1 Hypothesis: Animal welfare and animal health requirements for transport are complied with.

ransport route

Motor vehicle	1
aircraft	2
both	3

Transport via private individuals

Private individuals as flight sponsors	1
Private individuals/holidaymakers who take dogs by car	2
Exclusively cargo/commercial transport	3

If several answers apply, please enter several numbers in the field separated by a semicolon (;).

1.1.1. Violations of legal requirements (multiple answers possible)

Dogs are under 15 weeks old at the time of transport	1
Dangerous dogs are transported to Germany	2
The TRACES system is not used	3
Dog is not fit for transport for health reasons/ incurable diseases/ unhealed wounds, etc.	4

Please only consider the breeds Pit Bull Terrier, American Staffordshire Terrier, Staffordshire Bull Terrier and Bull Terrier and their crossbreeds.
The use of TRACES means an official veterinary examination before the start of transport and verification of identification, pet passport and rabies vaccination. This vet then makes a report to the European TRACES system, and the veterinary office at the destination automatically receives a report on the planned arrival of the dogs. The use of TRACES is mandatory for the commercial movement of dogs.

1.2. <u>Hypothesis: The organisation's work is transparent and traceable.</u>

Accommodation and quarantine options in Germany

Partner animal shelter in Germany	1
Association's own animal shelter in Germany	2
Foster home network in Germany	3
No accommodation facilities in Germany	0

What happens to dogs that are returned or require medical treatment before being rehomed? If several answers apply, please enter several numbers in the field separated by a semicolon (;).

Price for a dog = protection fee (interval-scaled)
If different values are given here depending on the category (male dog, female dog, castration yes/no, etc.) please enter the average value rounded to whole €.

1.2.1. <u>Hypothesis: There is comprehensive preparation and support for future owners.</u>

Detailed information about the dog is available.
Please always select the answer that applies to the majority of the dogs offered in this category.

1.2.1.1. Description of the character of the dogs

Yes	1
No	0

Please only indicate ‘yes’ here if the majority of the dogs on offer have such a detailed description that you can easily imagine the circumstances in which this dog might fit and what needs it might have of its future owners. A few words describing the character are not sufficient.

1.2.1.2. Photos of the dog

Yes	1

No	0

1.2.1.3. Videos oft he dog

Yes	1
No	0

1.2.2. An on-site pre-inspection is carried out

Yes	1
No	0

1.2.3. General information on the typical behaviour of foreign dogs is available

Yes	1
No	0

1.2.4. General information on typical diseases, in particular Mediterranean diseases/vector-borne diseases, is available on the homepage.

Yes	1
No	0

1.3. <u>Hypothesis: The claims made on the adopters are compliant with animal welfare legislation (TierSchG, HundehaltungsVO).</u>

1.3.1. *The following points should be based in particular on the adoption contract, if available.*

1.3.2. Contractual castration obligation

Yes	1
No	0

1.3.3. Regulations on the rejection of medical care (homeopaths etc.)

Yes	1
No	0

1.3.4. Training of the dog with methods contrary to animal welfare permitted

Yes	1
No	0

1.3.5. Use of accessories (collars, teletact, etc.) contrary to animal welfare.

Yes	1
No	0

1.3.6. Tethering/kennel husbandry without exercise possible

Yes	1
No	0
